# Modulation of Autophagy and mTOR pathways by Metformin in Olfactory Neuroepithelial Cells Across Different Age Groups: Insights into Pharmacological Implications for Neurodegeneration

**DOI:** 10.1002/alz.093304

**Published:** 2025-01-03

**Authors:** Maria‐del‐Carmen Cardenas‐Aguayo, Laura Gomez‐Virgilio, Obed‐Ricardo Lora‐Marin, Maria‐del‐Carmen Silva‐Lucero

**Affiliations:** ^1^ UNAM, School of Medicine, Department of Physiology, CDMX, DF Mexico

## Abstract

**Background:**

Longitudinal population‐based studies have consistently revealed an expedited cognitive decline in the elderly population with type 2 diabetes mellitus (DM2). Additionally, there is a documented increased risk of developing vascular dementia and Alzheimer’s disease in individuals with DM2. Conversely, recent research has pointed to metformin (MET), a widely prescribed medication for type 2 diabetes mellitus (T2DM), potentially mitigating age‐related cognitive dysfunction (Madhu et al., 2022). To explore this further, we utilized Neural Stem/Progenitor Cells derived from the Olfactory Epithelium (NS/PCs‐OE) obtained from healthy subjects across different age groups. The primary objective is to assess MET’s effectiveness in modulating mTOR signaling and enhancing autophagy, which likely could contribute to its observed positive effects on cognitive function.

**Method:**

To collect NS/PCs‐OE, volunteers were exfoliated from the anterior region of the medial‐lateral turbinate nasal regions, using a specific brush and circular movements. This study was authorized by the UNAM School of Medicine’s Ethical Committee (FMED/CEI/PMSS/074/2021). Participants gave written informed consent before nasal exfoliation. Immunodetection has been used to characterize those cell cultures. The cells were collected in DMEM/F‐12 supplemented with 4 mM L‐glutamine, 100 g/ml streptomycin, 100 IU/ml penicillin, and 10% fetal bovine serum. Cells from passages 5 through 10 from young and adult normal subjects were used in all experiments. We assessed the expression of p62, LC3, Lamp2a, Beclin‐1, and pmTOR with or without MET treatment by Western blotting. We also evaluate the expression of neuronal markers. Finally.

**Result:**

Human NS/PCs‐OE express proliferation (Ki67) and neural precursor (Nestin) markers. These cells also expressed βIII‐tubulin, but they did not express markers of mature neurons or glial markers. Moreover, human NS/PCs‐OE isolated from aged subjects show decreased autophagy and increased mTOR signaling in comparison with younger subjects. MET treatment enhanced the expression of autophagy markers. In addition, MET suppressed mTOR signaling.

**Conclusion:**

Our data showed that MET treatment inhibits mTOR signaling and enhances the autophagy pathway in NS/PCs‐OE from non‐diabetic aged subjects.

